# Traditional Dietary Pattern Increases Risk of Prostate Cancer in Argentina: Results of a Multilevel Modeling and Bias Analysis from a Case-Control Study

**DOI:** 10.1155/2015/179562

**Published:** 2015-11-16

**Authors:** Camila Niclis, María D. Román, Alberto R. Osella, Aldo R. Eynard, María del Pilar Díaz

**Affiliations:** ^1^Instituto de Investigaciones en Ciencias de la Salud, Consejo Nacional de Investigaciones Científicas y Técnicas, Universidad Nacional de Córdoba, Haya de la Torre Esquina Enfermera Gordillo, Ciudad Universitaria, 5016 Córdoba, Argentina; ^2^Escuela de Nutrición, Facultad de Ciencias Médicas, Universidad Nacional de Córdoba, Enrique Barros s/n, Ciudad Universitaria, 5016 Córdoba, Argentina; ^3^Laboratorio di Epidemiologia e Biostatistica, Istituto di Ricovero e Cura a Carattere Scientifico “Saverio de Bellis”, Via Turi 27, Castellana Grotte, 70013 Bari, Italy; ^4^Cátedra de Biología Celular, Histología y Embriología, Facultad de Ciencias Médicas, Universidad Nacional de Córdoba, Haya de la Torre Esquina Enfermera Gordillo, Ciudad Universitaria, 5016 Córdoba, Argentina

## Abstract

There is increasing evidence that dietary habits play a role in prostate cancer (PC) occurrence. Argentinean cancer risk studies require additional attention because of the singular dietary pattern of this population. A case-control study (147 PC cases, 300 controls) was conducted in Córdoba (Argentina) throughout 2008–2013. A principal component factor analysis was performed to identify dietary patterns. A mixed logistic regression model was applied, taking into account family history of cancer. Possible bias was evaluated by probabilistic bias analysis. Four dietary patterns were identified:* Traditional* (fatty red meats, offal, processed meat, starchy vegetables, added sugars and sweets, candies, fats, and vegetable oils),* Prudent* (nonstarchy vegetables, whole grains),* Carbohydrate* (sodas/juices and bakery products), and* Cheese* (cheeses). High adherence to the Traditional (OR 2.82, 95%CI: 1.569–5.099) and Carbohydrate Patterns (OR 2.14, 95%CI: 1.470–3.128) showed a promoting effect for PC, whereas the Prudent and Cheese Patterns were independent factors. PC occurrence was also associated with family history of PC. Bias adjusted ORs indicate that the validity of the present study is acceptable. High adherence to characteristic Argentinean dietary patterns was associated with increased PC risk. Our results incorporate original contributions to knowledge about scenarios in South American dietary patterns and PC occurrence.

## 1. Background

Changes in prostate cancer (PC) incidence of migrant populations [1] and geographical differences in PC incidence rates [2, 3] have motivated the study of possible lifestyle and environmental factors involved in the development of PC, including diet.

PC is the second most commonly diagnosed cancer among men globally. Among Argentinean men, PC is the most frequently diagnosed cancer and it is the third most common cause of cancer death [4].

However, the etiology of prostate carcinoma is mostly unknown and the role of dietary habits is rather controversial [5]. High intakes of some foods, such as dairy products, red meats, and processed meats, have been suggested as possible risk factors. Additionally, nutrients including *α*-linolenic acid and calcium seem to play a role in prostate carcinogenesis [6]. Despite the increasing number of published papers addressing the relationship between dietary habits and PC from different approaches, the issue is still open to discussion.

Due to the complexity of dietary intake and the potential for effect modifications among dietary components, a dietary eating patterns approach could be more suitable than the traditional analysis of isolated foods and nutrients [7]. Factor analysis has been broadly used in research into diet and PC associations in the last two decades to describe diet and disease associations [6]. Dietary patterns approach deals with the issue of collinearity of nutrients and possible interdependencies between foods and nutrients [8]. In addition, it simplifies the interpretation of a complex and multidimensional phenomenon such as dietary intake. Several studies have examined population dietary patterns related to PC in the last decade [9–13]. However, this strategy has not yet been addressed in Argentina for the study of PC. Traditionally, in populations of the region known as the Southern Cone (that includes Argentina, Uruguay, and Chile), the contribution of meat (especially red meat) to energy intake has been of considerable magnitude, providing in some cases about 50 percent of total daily energy [14, 15]. According to the FAO food balance sheets, in Argentina the per capita food supply of meat was 193 g/day in 2011, ranking first of all countries of America, while the United States comes on second place with 183 g/day [16]. Several observational studies found that higher intakes of total meat as well as red and processed meat were associated with the occurrence of PC when they were analyzed as an individual food group [6] or as a characteristic of a dietary pattern [12, 13]. Furthermore, and according to the global nutritional transition process, in the Southern Cone population there were changes in food consumption related to the inclusion of high-energy refined foods [14]. Consequently, additional attention must be paid to the study of cancer risk in this region due to its population's eating habits. Thus, it is necessary to consider the complex process of food consumption, intercrossed by many other cultural habit characteristics of subjects and populations.

The objective of this study was to estimate the effect of characteristic dietary patterns on the occurrence of PC in Argentinean men. Additionally, a sensibility analysis was conducted in order to obtain reliable estimations.

## 2. Methods

### 2.1. Design and Participants

The study was conducted within the framework of the Environmental Epidemiology of Cancer in Córdoba (EECC) project. In addition to case-control studies about dietary and other environmental exposures related to the cancers of highest incidence, the project includes the study of incidence analysis and spatial distribution and mortality trends and patterns.

This case-control study was conducted from January 2008 to December 2013 in Córdoba, the second most populated Argentinean province (3,067,000 inhabitants, according to the 2010 census), located in the center of the country. Cases were men with incident, histologically confirmed PC (ICD-10th Edition, ICIE10:C61) with no previous diagnosis of cancer in other sites. They were identified in public and private health institutions registered at the Córdoba Tumor Registry (CTR). Two controls per case, frequency matched by age (±5 years) and area of residence, were randomly chosen from the census list and included only after verifying the absence of any neoplastic or related condition as well as diseases or other conditions that generate long-term modifications to dietary habits. A total of 147 men with PC aged 48–89 (median age 72) and 300 controls aged 46–89 years (median age 71) were included. On average, 10% of cases and 10% of controls invited to take part in the interview refused to participate. Subjects interviewed were from rural (54%) and urban (46%) areas (including the most populated area, Córdoba City, with 1,300,000 inhabitants), in representative proportions of the total population of Córdoba province [4].

### 2.2. Subject Information

All participants were interviewed at home by centrally trained and routinely supervised nutritionists. A structured questionnaire was completed including information about sociodemographic characteristics, occupational history, smoking habits, alcohol consumption, self-reported anthropometric characteristics, physical activity, medical insurance, personal medical history, and family history of cancer. To assess dietary exposure, a validated food frequency questionnaire (FFQ) of 127 items [17] was completed. Subjects were asked about their dietary intake over the 5 years prior to diagnosis (cases) or interview (controls). The FFQ was coupled with an also validated photographical atlas based on standard portion sizes in Argentina [18]. The seasonal pattern of consumption of each vegetable or fruit was also taken into account. Physical activity was measured by means of the International Physical Activity Questionnaire [19]. Frequency and duration of physical activity were then expressed as metabolic equivalent of tasks (METs).

### 2.3. Dietary Pattern Identification

In the present work, a principal component factor analysis (PCFA) and a Varimax rotation method were applied on 300 male controls to characterization of dietary patterns. The food items contained in the dietary FFQ were classified into 24 predefined food groups based on similarities in the nutrient profile and culinary usage in the Argentinean diet [20]: milk/yogurt, cheese, lean red meat, fatty red meat, processed meat, offal, chicken, fish, eggs, fruits, nonstarchy vegetables, starchy vegetables, nuts, refined cereals, whole grains, bakery products, pulses, added sugar and sweets (sugar, jam, honey, and caramels), candies (*dulce de leche* (milk jam), ice cream, chocolates, and peanut butter), vegetable oils, fats, infusions, sugary drinks, and alcoholic drinks.

Factor analysis was then applied to reduce the food groups to a small number of factors that explained the maximum fraction of the variance [21]. The factorability of the correlation matrix was evaluated by applying the same criteria used previously [20]. To determine the number of components to be retained, the eigenvalues (greater than 1) and the Scree test were considered. Furthermore, the percentage of variance explained by each factor and the interpretability of the factors were taken into account. Each factor was named according to its dominant food groups and those with an absolute rotated factor loading ≥ 0.40 were considered. Each pattern was then correlated with life style and sociodemographic characteristics, using direct and partial correlation coefficients. As a second step, cases and controls were scored by applying the regression method. After that, all participants were categorized into quartiles of adherence to each factor score.

### 2.4. Risk Analysis

A multilevel logistic regression (MLR) model [22] for the binary response (1 if a case, 0 otherwise) was fitted. A hierarchical structure in the data was assumed: subjects (level 1), in order to assess individual-level variable effects such as dietary patterns to the outcome, clustered into a second level of aggregation, the family history of cancer, defined according three categories, first- or second-degree relatives with PC, first- or second-degree relatives with other cancer, or no family history of cancer. Identified dietary patterns, energy intake, body mass index (BMI), and occupational exposure (industrial exposure to chemical contaminants recognized by IARC as carcinogens, i.e., industries such as dyes, paints, textiles, plastics, rubber, leather, herbicides, automotive, chemical, and coal industry, for at least two years) were included as first-level covariates. A period of two years or more was considered because in the exploratory analysis higher risk was identified from this time onwards.

The median odds ratio (MOR) was calculated. MOR can be conceptualized as the increased risk that (in median) a subject would have if moving from one context of family history of cancer to another. In this study, MOR shows the extent to which the individual probability of having PC is determined by belonging to the family history of PC group. Additionally, the intraclass correlation coefficient (ICC) was also estimated. Akaike information criterion (AIC) was used to select the most suitable model [23].

### 2.5. Sensitivity Analysis

A multiple probabilistic sensitivity analysis was performed by assigning conventional probability density distributions to the values of the bias parameters [24]. Differential misclassification of exposure was assumed by drawing the sensitivities and specificities from different trapezoidal distributions for cases and controls. Minimum values equal to 0.70 and 0.75 and maximum ones equal to 0.90 and 1 were assigned in cases and controls specificity, respectively, while both sensitivities ranged from 0.75 to 1. Lower specificity in the cases group was assigned taking into account the possibility of recall bias.

Moreover, a higher probability to select unexposed cases and controls was assumed as respondents could have an increased interest in health-related issues and have healthier habits than nonrespondents. However, a small association between respondents-nonrespondents and PC is to be expected. Thus, we assigned a prior log-normal distribution to the selection-bias factor with mean 0 and standard deviation 0.21. This value of standard deviation is such that it permits the bias factor to fall 95% of times between 0.7 and 1.5 (exp(−1.96*∗*0.21) and exp(1.96*∗*0.21)), which yields 95% prior probability of the bias factor falling between exp(−1.96*∗*0.21) = 0.7 and exp(1.96*∗*0.21) = 1.5.

Finally, the potential confounding effect introduced by the effect of central obesity was considered as this condition could be associated with PC [25] and with a risky dietary pattern (such as the Traditional Pattern identified in this study). Thus, a prevalence of the confounder of 0.2 to 0.3 and 0.1 to 0.2 among those exposed and unexposed to Traditional Pattern was assigned, respectively. A log-normal prior probability distribution for the confounder-PC OR, with 95% confidence limits of ln(0.4) and ln(0.9), was specified. Thus, the mean of this prior distribution is {ln(0.4) + ln⁡(0.9)}/2 = −1.1268 with standard deviation {ln⁡(0.4) + ln⁡(0.9)}/(2*∗*1.96) = −0.0575. The multiple probabilistic sensitivity analysis was applied to the effect of Traditional Pattern on the risk of PC as it is the most characterizing pattern of the Argentinean diet [16, 20]. Stata 12.1 software was used for all statistical analysis [26].

## 3. Results

The characteristics of cases and controls are shown in Table 1. Both groups had a similar distribution of age, socioeconomic status, smoking habits, physical activity, energy intake, and BMI. On the other hand, occupational exposure and low educational level displayed higher percentages among cases compared with controls.

Factor loadings for food groups and the variance explained by each factor are shown in Table 2. Four distinct dietary patterns were identified from the factor analysis explaining 31.5% of total variance. The first factor, labeled* Traditional Pattern*, was positively loaded for fatty red meats, offal, processed meat, starchy vegetables, added sugars and sweets, candies, fats, and vegetable oils. The second factor, consisted of high loadings for nonstarchy vegetables, whole grains, and low loadings for alcoholic drinks, was named* Prudent Pattern*. The third factor, characterized by high loadings for sodas/juices and bakery products, was named* Carbohydrate Pattern*. The last factor was labeled* Cheese Pattern* because it was positively loaded for this food group and negatively loaded for fish. In this last pattern, even if both foods groups had similar loadings values, the name was chosen based on the food group more frequently consumed. The* Traditional Pattern* correlates strongly with total energy intake, intake of carbohydrates, lipids, cholesterol, calcium, and vitamin E (Table 3). Intake of carbohydrates was also strongly correlated with the* Carbohydrate Pattern*. The* Prudent Pattern* had negative correlations with ethanol (Table 3).

A higher score for the* Traditional Pattern* was associated with a lower proportion of men undertaking physical activity, while the inverse was found for the* Carbohydrate* (Table 4). Also, in higher quartiles for the Prudent Pattern as well as the* Cheese Pattern* a lower proportion of smokers was found. Distributions for the rest of the characteristics studied were similar across quartiles.

The* Traditional Pattern* and* Carbohydrate Pattern* were significantly associated with PC risk (OR 2.54; 95% CI 1.491–4.342 and OR 2.10; 95% CI 1.400–3.164, resp., quartile 1 as baseline), while the* Prudent *and* Cheese Patterns* were not significantly associated (OR 1.31; 95% CI 0.493–3.508 and OR 1.02; 95% CI 0.538–1.932, resp.) (Table 5).

Individual likelihood of PC occurrence was also dependent on family history of cancer hierarchy. It was observed that over 30% of the variance of the outcome is attributable to this clustering (ICC = 0.33). A MOR of 3.38 indicated that moving from no familiar history of cancer to a family history of PC increased by three times the individual odds of PC occurrence when randomly picking out two persons in different groups.

Besides, probabilistic sensitivity analysis showed that systematic and random error-adjusted median ORs (1.34) had slight differences with conventional ORs (1.33), and the ratio of 95% simulation limits including systematic and random error is nearly two times higher than the conventional one (Table 5).

## 4. Discussion

In total, four distinct dietary patterns in men participating in this study were identified; these were labeled* Traditional Pattern*,* Prudent Pattern*,* Carbohydrate Pattern*, and* Cheese Pattern*. The higher adherence to* Traditional *and* Carbohydrate Patterns* conferred an increased risk for PC. The* Prudent *and* Cheese Patterns* were not associated with PC risk. Moreover, there was a significant family history of cancer clustering effect.

The variance portions explained by each dietary pattern are similar to those reported in other studies of dietary patterns and PC which range from 1.72% to 11% [10, 11, 13].

Remarkably, two of the most characteristic patterns that emerged in this population had a promoting effect for PC occurrence. The pattern labeled* Traditional* was the most representative pattern and, with high loadings found for fatty red meats, offal, and processed meats, coincides with the main characteristics of Argentinean food habits described in previous studies [14, 15]. Frequently, patterns with red meat and/or processed meat and eggs in other studies were named “Western” (which include also sugar and candies), “Processed,” or “Carnic” patterns. In most cases high adherence to these patterns increased the risk of PC [10, 11, 13, 27, 28], in agreement with our results. Nevertheless, in other studies similar patterns do not show association with this disease [29, 30], including one of the largest studies published to date on dietary patterns and PC [31]. However, among men aged > 65 years, greater adherence to the Western pattern suggested an increased risk of PC in the aforementioned study.

The consumption of red meat in some countries of South America is among the highest in the world. Uruguay and Argentina rank first and second, respectively, with about 60 kg per year per capita [16]. Specifically in this study, subjects with higher adherence to the* Traditional Pattern* consumed a mean of 313 grams of fatty red meat daily, including the intake of offal, frequently added to the traditional Argentinean barbecue or* parrillada*. In fact, charcoal grilling is one of the most common methods for cooking meat in Argentina which results in a high formation of heterocyclic amines and polycyclic aromatic hydrocarbons, such as benzo(a)pyrene, both of which are considered carcinogens in animals [32]. Several case-control studies [33–37] have reported that meat overall and salted and red meat intake have a positive relation with PC, all of them showing ORs of 1.5 or higher.

The basis of the association between PC and high consumption of red meat is not known. An explanation considered is the high zinc content of red meat. This mineral is essential for testosterone synthesis and may have other effects on the prostate [37].

Additionally, meat is the major contributor to fat intake in the Southern Cone diet [15]. Thus, risk increase shown with high adherence to* Traditional Pattern* may reflect the high exposure to saturated fat from fatty meats and from fats, also present in this pattern. High fat intake (mainly saturated fatty acids and linoleic acid) appears to be associated with an increased risk of PC [6, 38]. Vegetable oil group characterizes this pattern as well. The main dietary oil consumed in this population is sunflower oil, which is predominantly a mixture of oleic (omega-9) and linoleic fatty acids (omega-6). However, Ma and Chapman [39], after reviewing numerous studies, concluded there was not enough evidence to draw conclusions about polyunsaturated fatty acid intake.

Also high intakes of eggs, starchy vegetables, and added sugar and sweets, the other food groups characterizing the* Traditional Pattern*, when analyzed separately were associated with a high risk of PC [40–44]. Nevertheless, the promoting effect for PC possibly results from the combination of food groups characterizing the* Traditional Pattern*. At the same time, this pattern is deficient in some anticarcinogenic dietary components. Some authors include the deficiency of vegetable foods intake as another hypothesis of high meat intake and PC association [37, 41].

The increasing risk of PC with high adherence to the* Carbohydrate Pattern* could be linked to the high carbohydrate content and glycemic index of food groups that characterize this pattern. Hyperglycemia induced by the intake of these beverages and foods stimulates high insulin secretions, which act* per se* as a growth factor and induce an increase of IGF-1 (insulin-like growth factor). IGF-1 stimulates anabolic metabolism, cell proliferation, and cell differentiation and can also inhibit apoptosis [44]. High intake of sodas, juices, sweets, added sugar, and other high glycemic index foods was associated with an increase in PC in other epidemiological studies [44, 45] whereas others found no associations [46, 47].

In most other studies, patterns comparable to the* Prudent Pattern* identified in the present study report similar results on PC risk. The Prudent Pattern in a prospective study using data from several cohort studies in the United States [28] showed no association with PC risk. Similar results were found in other studies regarding a Prudent Pattern (that in some cases also included dairy foods or fish) and its association with PC occurrence [10, 11, 13, 27, 29, 31]. Nonstarchy vegetables and fruits intake have a protective role for diverse tumors, possibly associated with a high content of antioxidant compounds, specially carotenoids and vitamins C and E [48]. However, the evidence in epidemiologic studies regarding vegetable intake and PC risk association is considered insufficient [5].

Diverse studies showed suggestive, but not definitive, evidence that dairy products, as well as the nutrients they provide, may increase the risk of PC [5, 49]. However, the* Cheese Pattern* was not associated with PC risk in this study. Cheeses are sometimes high in fat, animal protein, and calcium but also contain vitamin D and conjugated linoleic acid that may be protective [50–52]. Negative loading for fish of this factor also reflects the low intake of fish of Cordobesian population. About 30% of subject in this study did not consume fish, and the mean intake among those who do consume is 21 g/day (data not shown).

In the present study heterogeneity of responses coming from a second level of aggregation such as the family history of cancer was considered in the risk estimation process. Family history of cancer was selected based on the known heritability of this disease derived from either genetic susceptibility [42] or exposure to common environmental factors [53]. In accordance, the results showed a dependence on the PC risk linked to this clustering. Thus, the risk of PC in a subject without a family history of any cancer would increase if, given the same individual-level covariates, he had family history of PC.

Some issues concerning case-control studies limitations were considered in the analysis. Systematic errors are frequent in observational epidemiologic studies; however they seldom are measured quantitatively. In the present study the possibility of selection bias as well as recall bias, a classification bias caused by “rumination” in cases regarding the possible causes of their disease, was considered. Additionally, to avoid potentially important bias due to confounders, similar distribution of age and place of residence in cases and controls was sought, and both groups were interviewed in the same period. However, residual unmeasured confounders may be present, such as the presence of abdominal obesity, given its association with PC [25] and with a high adherence to a risky dietary pattern. ORs adjusted through quantitative bias analysis were slightly different compared with conventional ones. Therefore, the sensitivity analysis performed based on the possibility of systematic errors mentioned showed no major evidence of influence of bias.

FFQs may be prone to error; however the reproducibility of our 5-year FFQ has been accurately tested for epidemiological cancer studies [18]. This case-control study also benefits from the use of population rather than hospital based data, thus avoiding Berkson's bias (where hospital controls might not represent the prevalence of exposure in the community from which cases arise).

The use of the principal component analysis for identifying dietary patterns has potential strengths and limitations. In the first place, the labeling of the patterns is mainly subjective and derived from the authors' criteria. Besides, resulting patterns in a posteriori methodologies are specific to the population from which they emerge, which results in difficulty comparing with other studies. Even so, PCFA is the main statistical method proposed today to derive dietary patterns in cancer epidemiology research [8]. The study of information of multiple food intakes summarized in a unique exposition measurement constitutes a methodological advantage since it addresses the problem of multicollinearity and simplifies the interpretation of results [54].

Another aspect to consider is the small size of our study. Epidemiological and statistical literature provide asymptotic formulas for the computation of case-control sample sizes required for odds ratios, unadjusted or adjusted for a confounder [55]. However, all these recommendations only take into account fixed effects of covariates, including the intercept. The limited number of parameters imposed in the model (one for each pattern) and the constraint on the sources of variability (a variance component to quantify the intraclass correlation) constitute a suitable effort to compensate for the small size of our study.

The multilevel modeling approach constitutes a statistic and interpretative advantage as it proposes a theoretical construct for addressing diet-cancer relationship, based on an idea of reality organized hierarchically on dimensions (familial or contextual) [56, 57]. This is considered especially important in the study of health determinants, given that it provides relevant information that allows for assessing the importance of the context in different individual results in health [23].

## 5. Conclusion

The present work adds evidence about the effect of particular dietary patterns on PC occurrence, coupled with the association with the family history dimension. We concluded that the* Traditional* and the* Carbohydrate Patterns* could be associated with PC, possibly due to the presence of high loadings of fatty meats, eggs, starchy vegetables, and foods groups rich in sugar and fat, coupled with an absence of fresh vegetables, especially focusing on populations with a family history of PC. However, further studies are needed for strong statements regarding the etiology of PC.

## Figures and Tables

**Table 1:** Characteristics of cases and controls, Córdoba, Argentina (2008–2012).

	Cases (*n* = 147) *n* (%)	Controls (*n* = 300) *n* (%)
Age (years)		
≤60	17 (11.56)	40 (13.33)
61–70	48 (32.65)	109 (36.33)
71–80	63 (42.86)	115 (38.33)
≥81	19 (12.93)	36 (12.00)
Socioeconomic status		
Low	61 (41.50)	114 (38.00)
Middle	50 (34.01)	103 (34.33)
High	36 (24.49)	83 (27.67)
Educational level		
Low	34 (23.13)^*∗*^	47 (15.82)^*∗*^
Middle	70 (47.62)	141 (47.47)
High	43 (29.25)	109 (36.70)
Occupational exposure^a^		
No	99 (67.35)	219 (73.24)
Yes	48 (32.65)^*∗*^	80 (26.76)^*∗*^
Smoking habits		
No	51 (34.69)	92 (30.67)
Yes	96 (65.31)	208 (69.33)
Lifetime physical activity		
Low	79 (53.74)	178 (59.33)
Middle	51 (34.69)	100 (33.33)
High	17 (11.56)	22 (7.33)
BMI		
≤24.9	34 (24.44)	84 (27.66)
25–29.9	87 (57.78)	150 (49.65)
≥30	26 (17.78)	66 (22.70)
Energy intake^b^		
Low	40 (27.21)	100 (33.33)
Middle	48 (32.65)	100 (33.33)
High	59 (40.14)	100 (33.33)

^*∗*^Proportion values significantly different (*p* ≤ 0.1). ^a^Exposure to chemical contaminants for 2 years or longer. ^b^Categories based on tertiles of intake in controls.

**Table 2:** Factor loading matrix for dietary patterns, Córdoba, Argentina (2008–2012).

Food Groups	Traditional Pattern	Prudent Pattern	Carbohydrate Pattern	Cheese Pattern
Milk/yogurt	0.1762	0.1780	0.1496	0.3391
Cheese	0.2212	0.2715	−0.2937	**0.4665**
Lean red meat	0.3395	−0.1049	−0.2270	0.1403
Fatty red meat	**0.4868**	−0.1796	0.0283	−0.2882
Offal	**0.4355**	−0.2090	−0.0477	−0.3108
Processed meat	**0.4316**	−0.0768	−0.0843	−0.1595
Chicken	0.1273	0.2138	0.3717	−0.1484
Fish	0.0390	0.3644	0.1642	**−0.4637**
Eggs	0.3913	−0.1943	−0.0578	−0.0988
Fruit	0.1603	0.2817	−0.3770	0.2776
Nonstarchy vegetables	0.3303	**0.4638**	−0.1302	0.1328
Starchy vegetables	**0.5169**	0.1854	−0.2335	−0.1884
Nuts	0.1136	0.1399	0.2209	0.0656
Refined cereals	0.3032	−0.3946	0.0874	0.2006
Whole grains	−0.0454	**0.5012**	−0.2888	−0.1246
Bakery products	0.2489	−0.1775	**0.4549**	0.1017
Pulses	−0.1131	0.1059	0.1264	−0.1407
Added sugar and sweets	**0.5128**	−0.2968	0.2170	0.2944
Candies	**0.4152**	0.2613	0.3331	0.2706
Fats	**0.4348**	0.3442	0.2577	−0.4030
Vegetable oils	**0.5967**	0.1190	−0.0961	−0.0144
Infusions	0.3032	0.0261	−0.2030	0.2505
Alcoholic drinks	0.3357	**−0.4868**	−0.2894	−0.1957
Sugary drinks	0.1032	0.1061	**0.5419**	0.2183
*% Total variance explained*	11.81	7.29	6.53	5.89
*% Total variance explained accumulated*	11.81	19.10	25.63	31.52

Note: food groups with factor loadings ≥0.40 are in bold.

**Table 3:** Pearson correlations between dietary pattern scores and key nutrients, Córdoba, Argentina (2008–2012).

Nutrient	Simple correlations				Partial correlations			
	Traditional Pattern	Prudent Pattern	Carbohydrate Pattern	Cheese Pattern	Traditional Pattern	Prudent Pattern	Carbohydrate Pattern	Cheese Pattern
Energy (Kcal)	0.7523^*∗*^	−0.0382	0.2588^*∗*^	−0.1445^*∗*^	0.1577^*∗*^	−0.0990	0.0829^*∗*^	−0.1211^*∗*^
Carbohydrates (g)	0.6677^*∗*^	−0.0666	0.4921^*∗*^	0.3358^*∗*^	−0.0987	0.0621	0.2709^*∗*^	−0.0657
Proteins (g)	0.3934^*∗*^	0.1540^*∗*^	0.2645^*∗*^	−0.2198^*∗*^	−0.2934^*∗*^	0.1456^*∗*^	−0.0321^*∗*^	0.0479
Lipids (g)	0.6267^*∗*^	0.0672	0.2175^*∗*^	−0.3386^*∗*^	−0.1101	0.1009	0.0504^*∗*^	0.0191
Cholesterol (mg)	0.5419^*∗*^	0.0001	0.2059^*∗*^	−0.3516^*∗*^	0.4197^*∗*^	−0.2095^*∗*^	0.2949	0.0181^*∗*^
Calcium (mg)	0.4181^*∗*^	0.3318^*∗*^	−0.0088	0.3349^*∗*^	0.2599^*∗*^	0.3792^*∗*^	−0.2610^*∗*^	0.0557^*∗*^
Vitamin A (mg)	0.2771^*∗*^	0.3855^*∗*^	−0.1019	−0.0198	0.2753^*∗*^	0.3306^*∗*^	−0.2567	0.0670
Vitamin E (mcg)	0.6222^*∗*^	0.1150^*∗*^	−0.1126	0.0678	0.4721^*∗*^	0.1551^*∗*^	−0.2846^*∗*^	−0.0564^*∗*^
Selenium (mcg)	0.3727^*∗*^	0.2498^*∗*^	0.0828	−0.2561^*∗*^	0.0533	0.3141^*∗*^	−0.0416	−0.3760^*∗*^
Ethanol (g)	0.2662^*∗*^	−0.4493^*∗*^	−0.2416^*∗*^	−0.2033^*∗*^	−0.1135	−0.0180	−0.1921^*∗*^	0.4194

^*∗*^ *p* ≤ 0.05.

**Table 4:** Personal characteristics by quartiles of dietary pattern scores, Córdoba, Argentina (2008–2012).

		Quartiles of dietary pattern scores			
		I	II	III	IV
Traditional Pattern		*n* = 99	*n* = 114	*n* = 112	*n* = 122
Age	Mean (SD)	71.26 (8.86)	71.38 (8.34)	70.04 (8.08)	69.61 (69.61)
Current BMI		27.16 (3.22)	27.51 (3.66)	27.05 (3.63)	27.61 (3.76)
Usual BMI		27.15 (3.33)	27.45 (3.68)	27.09 (3.76)	27.45 (3.99)
Smoker	%	73	64	68	68
Family history of PC		9	11	8	8
Occupational exposure^a^		20	34	33	26
Vigorous or moderate PA^b^		54	54	46	35

Prudent Pattern		*n* = 116	*n* = 103	*n* = 109	*n* = 119
Age	Mean (SD)	68.94 (8.44)	69.88 (8.49)	71.64 (8.05)	71.64 (8.77)
Current BMI		27.83 (3.59)	27.58 (3.47)	27.15 (3.70)	26.85 (3.54)
Usual BMI		27.41 (3.86)	27.76 (3.90)	27.01 (3.55)	27.04 (3.53)
Smoker	%	80	70	67	55
Family history of PC		10	11	6	9
Occupational exposure^a^		25	32	29	29
Vigorous or moderate PA^b^		53	50	43	41

Carbohydrate Pattern		*n* = 97	*n* = 111	*n* = 123	*n* = 116
Age	Mean (SD)	70.42 (9.29)	70.60 (8.56)	71.06 (8.11)	70.01 (8.24)
Current BMI		27.87 (3.83)	27.36 (3.45)	26.70 (3.24)	27.57 (3.78)
Usual BMI		27.59 (3.98)	26.89 (3.09)	27.01 (3.20)	27.73 (4.43)
Smoker	%	70	64	70	68
Family history of PC		7	13	9	8
Occupational exposure^a^		24	26	32	32
Vigorous or moderate PA^b^		37	41	55	52

Cheese Pattern		*n* = 108	*n* = 115	*n* = 112	*n* = 112
Age	Mean (SD)	69.49 (9.00)	69.90 (7.76)	71.12 (8.7)	71.62 (8.48)
Current BMI		27.89 (4.01)	27.40 (3.30)	27.23 (3.66)	26.89 (3.32)
Usual BMI		27.48 (3.80)	27.21 (3.33)	27.32 (4.32)	27.18 (3.37)
Smoker	%	74	69	68	62
Family history of PC		13	10	5	9
Occupational exposure^a^		26	26	28	35
Vigorous or moderate PA^b^		41	55	46	46

SD, standard deviation; BMI, body mass index; PC, prostate cancer; PA, physical activity. ^a^Exposure to chemical contaminants for 2 years or longer. ^b^ Subjects who performed regular physical activity reaching at least 600 METs by minutes/week.

**(a):** 

	Number of cases	OR (CI 95%)^a^	*p* value	*p* for trend
Traditional Pattern				
Quartile I	24	1	—	0.048
Quartile II	39	1.60 (0.970–2.660)	0.065	
Quartile III	37	1.73 (1.167–2.575)	0.006	
Quartile IV	47	2.54 (1.491–4.342)	0.001	
Prudent Pattern				
Quartile I	41	1	—	0.926
Quartile II	28	0.70 (0.396–1.264)	0.243	
Quartile III	34	0.84 (0.540–1.310)	0.445	
Quartile IV	44	1.31 (0.493–3.508)	0.584	
Carbohydrate Pattern				
Quartile I	22	1	—	0.069
Quartile II	36	1.76 (1.254–2.479)	0.001	
Quartile III	48	2.67 (0.975–7.349)	0.056	
Quartile IV	41	2.10 (1.400–3.164)	<0.001	
Cheese Pattern				
Quartile I	33	1	—	0.720
Quartile II	40	1.48 (0.690–3.202)	0.310	
Quartile III	37	1.34 (0.842–2.155)	0.213	
Quartile IV	37	1.02 (0.538–1.932)	0.950	

**(b):** 

Bias analysis ORs	Percentiles	Ratio		
	2.5	50	97.5	2.5/97.5
Conventional	0.90	1.33	1.98	2.21
Systematic error	0.80	1.34	2.27	2.83
Systematic and random error	0.70	1.34	2.58	3.69

OR, odds ratio; CI, confidence interval. ^a^Age, BMI, energy intake, and occupational exposure were included in the MLR as covariates at first level, and family history of cancer was included at second level (variance 1.637, standard error 0.099, intraclass correlation coefficient 0.33, and median odds ratio 3.38).
